# Application of “Internet +” continuous emotion management training in patients with depression

**DOI:** 10.3389/fpsyt.2024.1452717

**Published:** 2024-11-26

**Authors:** Yanping Zhang, Jiaxin Wang, Linlin Qiao, Yating Li, Runing Hou, Xiaojing Gu, Jingyuan Zhao, Fang Yan

**Affiliations:** ^1^ Department of Psychiatry 7, The Second Affiliated Hospital of Xinxiang Medical University, Xinxiang, China; ^2^ School of Nursing, Xinxiang Medical University, Xinxiang, China; ^3^ Department of Mood Disorders 1, The Second Affiliated Hospital of Xinxiang Medical University, Xinxiang, China; ^4^ Henan Collaborative Innovation Center of Prevention and Treatment of Mental Disorder, Department of Nursing, The Second Affiliated Hospital of Xinxiang Medical University, Xinxiang, China

**Keywords:** internet intervention, continuity service, emotion management training, depression, nursing

## Abstract

**Objective:**

Explore the application effects of “Internet +” continuous emotional management training in depression patients and analyze the feasibility of innovative rehabilitation training methods.

**Methods:**

Conveniently selected 100 discharged patients meeting the diagnostic criteria for depression from the Second Affiliated Hospital of Xinxiang Medical University between August 2022 and January 2023 as study subjects. They were divided into an intervention group and a control group according to their discharge time, with 50 patients in each group. The control group received routine monthly telephone follow-ups and “Internet +” health popularization. The intervention group received “Internet +” continuous emotional management training in addition to the control group’s protocol. Before and after the intervention, the Hamilton Depression Rating Scale (HAMD), the Chinese version of the Cognitive Emotion Regulation Questionnaire (CERQ-C), and the Social Adaptation Function Evaluation Scale (SAFE) were used to compare the differences in depression relief, cognitive emotion regulation levels, and social functions between the two groups.

**Results:**

Two patients in the control group and three patients in the intervention group were lost to follow-up during the intervention, Ultimately, 48 patients in the control group and 47 patients in the intervention group completed the study. Before the intervention, there were no statistically significant differences between the two groups in HAMD scores, CERQ-C subscale scores, and SAFE scores (*P* < 0.05). After the intervention, the intervention group had significantly lower HAMD and SAFE scores compared to the control group (*P* < 0.05). In the CERQ-C questionnaire, the intervention group scored lower in self-blame, rumination, catastrophizing, and blaming others but higher in acceptance, positive refocusing, refocusing on planning, positive reappraisal, and putting into perspective compared to the control group, with statistically significant differences (*P* < 0.05).

**Conclusion:**

“Internet +” continuity emotion management training can reduce the severity of depression in post-discharge patients and enhance their emotional management and social adaptability. This approach is feasible in clinical practice.

## 1 Introduction

Depression is a common and severe psychological disorder characterized mainly by significant and persistent low mood, typically manifesting between the ages of 20 and 30. It poses substantial distress and burden on patients’ lives and works (1–4). The lifetime prevalence of depression in China is 6.8%, among which two-thirds of patients have had thoughts of suicide, with 15% ultimately choosing to end their lives through suicide (5). Every year, the number of deaths attributed to suicide related to depression reaches as high as one million (6). Depression treatment emphasizes comprehensive management throughout the entire course of the illness, with the ultimate goal being the restoration of social functioning (5, 7, 8). Multiple studies indicate that effective emotional management in patients suffering from depression can enhance their social functioning and foster positive beliefs (9–11). The emotional ABC theory in continuous emotional management training is a targeted psychotherapeutic approach for addressing psychological issues in groups with negative emotions (12, 13). Its unique advantage lies in examining and disputing the irrational beliefs underlying the negative emotions of depression patients, helping them establish rational beliefs and thinking, thereby achieving positive emotions and behaviors (14–17). The “Internet +” approach enables depression patients to engage in emotion management training programs regardless of location, facilitating communication with professionals and providing them with real-time interactive channels. When patients experience emotional fluctuations or encounter issues during the intervention process, they can seek help and receive feedback from professionals at any time. By intervening through the Internet, patients can quickly obtain support and guidance, which helps alleviate anxiety and enhance their confidence in recovering from depression. The “Internet +” emotional ABC theory is considered a future direction for the psychological treatment of patients with cancer and depression due to its high efficiency, ease of implementation, and broad impact (10, 18, 19). This study utilizes the “Henan Province Psychological Assistance Cloud Platform” at the Second Affiliated Hospital of Xinxiang Medical University, employing “Internet +” continuous emotional management training to compare changes in depression, cognitive emotional regulation, and social function recovery between two groups of patients. It aims to provide new interventions for post-discharge continuous care for depression patients and contribute to the advancement of mental health services.

## 2 Subjects and methods

### 2.1 Study subjects

A convenient sample of depression patients discharged from the Second Affiliated Hospital of Xinxiang Medical University between August 2022 and January 2023 were selected as study subjects and divided into intervention group and control group based on discharge time. Inclusion Criteria: (1) Meets the diagnostic criteria for depressive episodes and recurrent depressive disorder according to the International Classification of Diseases 10th Revision (ICD-10) (9); (2) According to Davis JM’s cut-off, scores ≤ 35 on the 24-item Hamilton Depression Scale (HAMD) (10), indicating moderate or mild depression; (3) Age between 18 and 60 years; (4) At least primary school education, capable of normal communication and interaction; (5) Proficiency in using computer or mobile information systems; (6) Informed consent from patients and their families, voluntarily participating in this study. Exclusion Criteria: (1) Patients with intellectual disabilities or psychiatric symptoms; (2) Patients with other comorbid physical diseases; (3) Patients unable to use computer or mobile information systems. This study has been approved by the Ethics Committee of the Second Affiliated Hospital of Xinxiang Medical University, Approval Number: XYEFYLL-(Scientific Research)-2022-48.

### 2.2 Research methods

#### 2.2.1 Intervention methods for the experimental group

##### 2.2.1.1 Establishment of the “Internet +” continuity emotional management training team

All team members hold a medical license or psychiatric nurse license and are staff members of our hospital. The team includes one head nurse from each of the male and female wards, five psychiatric rehabilitation therapists authorized by the hospital’s Medical Technology Clinical Application Committee, five psychiatric nurses (assistants), and five psychiatrists, totaling 17 members. The team is divided into five training groups, each consisting of one psychiatric rehabilitation therapist, one psychiatric nurse, and one psychiatrist. Each group is responsible for 10 patients. The psychiatric rehabilitation therapist conducts emotional management training for depression patients every Friday from 19:30 to 20:30. The training consists of 10 sessions per course. After the training concludes, the psychiatric rehabilitation therapist provides a summary to reinforce the acquired knowledge and asks each patient to write a reflection on their experience. The group psychological counseling process and main topics based on the ABC theory of emotional management are detailed in **Table 1**. Psychiatric nurses assist the psychiatric rehabilitation therapist during the training to ensure intervention effectiveness, conduct follow-up phone calls, and disseminate “Internet +” health popularization. Psychiatrists are responsible for selecting research subjects, diagnosing diseases, prescribing medications, and conducting scale assessments. The intervention group accesses the Henan Province Psychological Assistance Cloud Platform via computer or mobile phone for video-based sessions. The platform is centrally managed by information personnel and operates on an appointment basis. Two reminders are given 10 minutes before the start of each session. After clicking “Start Training,” participants can select “Emotional Management Training” to begin. Psychiatric rehabilitation therapists set permissions according to the course schedule to protect patient privacy and ensure the security of patient information. Training is conducted in an online video format.

**Table 1:** Content of continuous emotional management training.

Theme	Objective	Content	Intervention
A: Triggering events	Learning to identify the causes or triggers of events	Lesson 1: Warm-up games to create a positive psychological atmosphere, stimulate participants’ enthusiasm, and encourage openness, laying the foundation for training. Briefly introduce the course plan. Lesson 2: Explain the ABC theory and conduct exercises. ① Use video playback, role-playing, and real-life event descriptions to help participants identify the causes of triggering events; ② Engage in debates about rational and irrational beliefs in these events; ③ Discuss and summarize the rational and irrational beliefs present in the events.	Video streaming Role-playing Discussion summary
B: Beliefs	Developing rational beliefs	Lesson 1: Learning to shift attention; helping patients transform negative emotions into positive ones: ① When feeling distressed, troubled, or down, redirect attention to activities and topics of interest; ② Change the environment: such as going for a walk, traveling, or changing the place of residence. Lesson 2: Practicing attention-shifting to foster rational beliefs in patients: ① Help patients discard irrational, absolutist, and catastrophic beliefs; ② Assist patients in developing rational beliefs about certain events; ③ Guide patients in exploring their attitudes, feelings, values, and behaviors; ④ Aid patients in deepening self-awareness and understanding and accepting others.	Group instruction Simulation training Discussion analysis
		Lesson 1: Learning to appropriately express emotions; alleviating negative emotions through suitable methods in appropriate settings, such as crying, talking, or exercising. ① Articulate thoughts clearly and sharply; ② Share feelings with family or friends; ③ Release emotions through physical activities or labor; ④ Shout in open spaces like forests or fields. Lesson 2: Practicing appropriate emotional expression to foster rational beliefs in patients (same as above).	Group instruction Simulation training Discussion analysis
		Lesson 1: Learning positive self-suggestion; using stories to help patients replace outdated, negative thought patterns with more positive ideas and concepts, such as “Victory and defeat are common in battle” and “A blessing in disguise.” Lesson 2: Practicing positive self-suggestion to foster rational beliefs in patients (same as above).	Group instruction Simulation training Discussion analysis
C: Outcomes or responses	Altering emotional responses	Lesson 1: Learning social interaction regulation; helping patients set long-term and short-term goals, and encouraging them to seek out friends and family for interaction and conversation when troubled. Learning emotional sublimation and practicing the redirection of negative emotions toward self-beneficial and socially beneficial directions, with consultation and sharing with family or friends. Lesson 2: ① Help patients develop new cognitions; ② Assist patients in applying learned concepts in real life; ③ Encourage patients to express their experiences and exchange with others; ④ Review and consolidate learned knowledge, and have patients write reflections on their training experience.	Group instruction Simulation training Reflection writing

#### 2.2.2 Control group intervention methods

Patients receive regular medication treatment and monthly follow-up calls from a psychiatric nurse, lasting approximately 10-15 minutes. Follow-up includes medication adherence and stability of the condition. Additionally, patients access the Henan Province Psychological Assistance Cloud Platform, which weekly updates and sends “Internet +” health education materials, covering topics such as maintaining good sleep, emotional well-being, and coping with stress.

### 2.3 Evaluation metrics

A. General Information: Patient demographic survey, including gender, age, education level, occupation, marital status, illness duration, and number of hospitalizations.

B. The Hamilton Depression Scale (HAMD), developed by Hamilton (20), is used to evaluate the severity of depression in patients and is suitable for assessing depressive symptoms in adult patients. It is widely used in psychiatry, where psychiatrists assess patients through interviews. The scale comprises 24 items, with higher scores indicating more severe depression.

C. The Chinese version of the Cognitive Emotion Regulation Questionnaire (CERQ-C) is used to assess patients’ cognitive emotional levels and strategies after experiencing negative life events (21–23).

The questionnaire includes 36 items across 9 dimensions: self-blame, rumination, acceptance, catastrophizing, blaming others, positive refocusing, refocusing on planning, positive reappraisal, and putting into perspective. Acceptance, positive refocusing, positive reappraisal, refocusing on planning, and putting into perspective are adaptive strategies, while self-blame, rumination, catastrophizing, and blaming others are maladaptive strategies. The Cronbach’s *α* coefficient for the total scale is 0.81, and subscales range from 0.48 to 0.91. The retest reliability for the total scale is 0.56, and subscales range from 0.36 to 0.69. The average inter-item correlation for the total scale is 0.10, and subscales range from 0.19 to 0.71. Higher scores in each dimension indicate a greater tendency toward that particular emotion regulation strategy.

D. The Social Adaptive Functioning Evaluation (SAFE), developed by Guizhong Yao et al. (24) is utilized by psychiatrists through interviews and observation to assess the social adaptive functioning of depression patients over 18 years old. It includes 19 items across four dimensions: basic living skills, advanced living skills, social skills, and communication skills, effectively distinguishing levels of social adaptive functioning. Higher scores indicate greater impairment. The scale’s test-retest reliability is 0.96-0.99, inter-rater reliability is 0.77, and internal consistency ranges from 0.77 to 0.95.

### 2.4 Data collection methods

Data collection is conducted by psychiatric nurses from the research team. General information is gathered via the hospital information system, including patient demographics such as gender, age, education level, occupation, marital status, illness duration, and number of hospitalizations. HAMD, CERQ-C, and SAFE assessments are performed on both intervention and control group patients on the day of discharge and within one week after completing the training.

### 2.5 Quality control

(1) Implementation follows the “Rehabilitation Training Guidelines for Psychiatric Patients” established by the Second Affiliated Hospital of Xinxiang Medical University, with standardized training by rehabilitation therapists.
(2) The head nurse schedules the participating medical staff in advance and provides appropriate compensatory rest according to their working hours.
(3) Absence from training sessions twice or more is considered a dropout.

### 2.6 Statistical methods

Data entry and organization were performed using Excel 2019, with double-checking by two individuals. Statistical analysis was conducted using SPSS 27.0. Normally distributed continuous data were described by mean and standard deviation, and inter-group comparisons were made using independent samples t-test. Non-normally distributed continuous data were described by median and interquartile range, and inter-group comparisons were conducted using the Mann-Whitney U test. Categorical data were presented as frequencies and percentages, with inter-group comparisons made using the chi-square test. A P-value of less than 0.05 was considered statistically significant.

## 3 Results

### 3.1 Baseline characteristics of patients

A total of 48 patients in the control group (2 dropped out) and 47 patients in the intervention group completed the training (1 missed two sessions, 2 withdrew voluntarily). The baseline characteristics of the two groups are presented in **Table 2**.

**Table 2:** Comparison of general characteristics between the two groups. Unit: Cases(%).

Item	Category	Intervention group (n=47)	Control group (n=48)	Statistical Value	*P*
Gender	Male Female	26(55.3) 21(44.7)	23(47.9) 25(52.1)	*χ* ^2^ = 0.521	0.470
Age		36.93 + 6.41	36.25 + 6.19	*t* = 0.530	0.597
Educational Level	Middle school or lower High school or vocational school	3(6.4) 16(34.0)	2(4.2) 14(29.2)	*z* = 1.513	0.130
	Associate degree Bachelor’s degree or higher	24(51.1) 4(8.5)	20(41.7) 12(2.5)		
Occupation	Student Farmer Employee Others	1(2.1) 18(38.3) 16(34.1) 12(25.5)	0(0.00) 24(50.0) 15(31.2) 9(18.8)	*χ* ^2^ = 2.308	0.511
Marital Status	Unmarried Married Divorced	11(23.4) 25(53.2) 11(23.4)	14(29.2) 26(54.2) 8(16.7)	*χ* ^2^ = 0.843	0.656
Duration of Illness (years)	< 2	14(29.8)	19(39.6)	*χ* ^2^ = 1.029	0.598
	2~10	21(44.7)	19(39.6)		
	> 10	12(25.5)	10(20.8)		
Number of Hospitalizations	1	15(31.9)	16(33.3)	*z* = 0.137	0.891
	2~3 >3	29(61.7) 3(6.4)	27(56.3) 5(10.4)		

### 3.2 Comparison of HAMD scores between the two groups

Normality and homogeneity of variances for HAMD scores before and after the intervention in both groups were tested using the W test and Levene’s test. Results indicated that HAMD scores were normally distributed and had equal variances. Consequently, an independent samples t-test was used to compare HAMD scores before and after the intervention between the two groups, revealing statistically significant differences (*P* < 0.05). See **Table 3**.

**Table 3:** Comparison of HAMD scores between the two groups [Points, Mean ± SD].

Group	Before intervention	After intervention
Intervention group (*n* = 47)	18.27 ± 4.51	15.63 ± 3.08
Control group (*n* = 48)	18.08 ± 3.25	17.47 ± 3.38
*t*	0.240	-2.768
*P*	0.811	0.007

### 3.3 Comparison of CERQ-C scores between the two groups

Normality and homogeneity of variances for CERQ-C questionnaire dimension scores before and after the intervention in both groups were tested using the W test and Levene’s test. Results indicated that CERQ-C dimension scores were not normally distributed and had unequal variances. Therefore, the Mann-Whitney U test was used to compare CERQ-C dimension scores before and after the intervention between the two groups. The results showed that the intervention group had lower scores in self-blame, rumination, catastrophizing, and blaming others, and higher scores in acceptance, positive refocusing, refocus on planning, positive reappraisal, and putting into perspective compared to the control group, with statistically significant differences (*P* < 0.05). See **Table 4**.

**Table 4:** Comparison of CERQ-C scores between the two groups [Points, M (P25, P75)].

Project	Group	Before intervention	After intervention
Self-blame	Intervention group (n = 47) Control group(n = 48) *z* *P*	12 (10,13) 12 (10,13) -0.053 0.958	8 (7,9) 11.5 (9,13) -4.176 < 0.001
Acceptance	Intervention group (n = 47) Control group(n = 48) *z* *P*	8 (7,9) 8 (7,9) -0.319 0.750	12 (10, 13) 8 (7,9) -6.009 < 0.001
Rumination	Intervention group (n = 47) Control group(n = 48) *z* *P*	12 (11, 13) 12 (10, 13) 0.023 0.982	8 (7,9) 12 (10,13) -5,525 < 0.001
Positive refocus	Intervention group (n = 47) Control group(n = 48) *z* *P*	6 (5,8) 6 (5,8) -1.387 0.165	10 (7,13) 7 (6,8) -4.209 < 0.001
Refocus on planning	Intervention group (n = 47) Control group(n = 48) *z* *P*	5 (5.7) 6 (5.7) -1.195 0.232	12 (10, 13) 7.5 (7,8) -5.910 < 0.001
Positive reappraisal	Intervention group (n = 47) Control group(n = 48) *z* *P*	5 (5.7) 5 (5.7) -1.317 0.188	10 (7,13) 7 (6,8) -4.332 < 0.001
putting into perspective	Intervention group (n = 47) Control group(n = 48) *z* *P*	6 (5,7) 6 (5.7) -0.695 0.487	11 (7,13) 7 (6,8) -5.050 < 0.001
Catastrophizing	Intervention group (n=47) Control group(n = 48) *z* *P*	12 (10, 13) 12 (10, 13) -0.170 0.865	8 (7,8) 11 (10,13) -6.769 < 0.001
Blaming others	Intervention group (n = 47) Control group(n = 48) *z* *P*	12 (10, 13) 12 (10, 13) -0.323 0.746	8 (6,9) 12 (10,13) -4.849 < 0.001

### 3.4 Comparison of SAFE scores between the two groups

Normality and homogeneity of variances for SAFE scores before and after the intervention in both groups were tested using the W test and Levene’s test. Results indicated that SAFE scores were normally distributed and had equal variances. Therefore, an independent samples t-test was used to compare SAFE scores before and after the intervention between the two groups. The results showed statistically significant differences (*P* < 0.05). See **Table 5**.

**Table 5:** Comparison of SAFE scores between the two groups [Points, Mean ± SD].

Group	Before intervention	After intervention
Intervention group (*n* = 47)	16.42 ± 1.59	10.68 ± 1.21
Control group(*n* = 48)	16.47 ± 1.61	15.89 ± 1.85
*t*	-0.163	-16.136
*P*	0.871	< 0.001

## 4 Discussion

### 4.1 “Internet +” continuous emotional management training can alleviate depression in patients with depression

Patients with depression primarily exhibit low mood, loss of the ability to experience pleasure, self-blame, and guilt (25–27). They often adopt a negative thinking pattern, holding a pessimistic view of themselves, the world, and the future (28–31). Alenezi’s study indicates a significant negative correlation between the severity of depression and emotional regulation in these patients (32). The results of this study show that the HAMD depression scale scores of the intervention group were lower than those of the control group (*P* < 0.05). The reason for this may be that emotional management training by psychiatric rehabilitation therapists helps patients realize that low mood, distress, and other negative experiences are not caused by external stimuli, but rather by their perceptions of these stimuli. The same event can lead to different emotional responses based on differing cognitions and perceptions. Patients are assisted in simulating how to express and release emotions, choosing methods tailored to their preferences and characteristics, such as talking, exercising, etc. They are advised to take deep breaths at first and then shift their attention when unable to control emotions, for example, by listening to music or taking a walk.

Once their emotions are stable, analyze and discuss the causes of their mood decline, allowing them to express their inner feelings. Assist them in venting their depressive emotions using appropriate methods and techniques, guiding them to recognize and attempt to accept these emotions rather than avoiding or suppressing them, thereby alleviating depressive symptoms. Kreibig’s research suggests that emotion regulation reduces negative emotional responses and increases positive ones (33). “Internet +” continuous emotional management training can help patients build a support network by providing understanding and support from psychiatrists, psychiatric nurses, and psychiatric rehabilitation therapists. Additionally, “Internet +” enhances patient engagement in training, aiding in coping with depression and alleviating depressive symptoms. Keeler’s research indicates that online interventions can reduce depression severity and improve treatment adherence in patients (34), consistent with the findings of this study.

### 4.2 “Internet +” continuous emotional management training enhances emotional regulation in depression patients

This study shows that in the intervention group, the CERQ-C adaptive strategies of acceptance, positive refocusing, refocus on planning, positive reappraisal, and put into perspective scores were significantly higher than those of the control group (*P* < 0.05). In the questionnaire, the maladaptive strategies of self-blame, rumination, catastrophizing, and blaming others were significantly lower in the intervention group compared to the control group (*P* < 0.05). The possible reason for this is that during training, psychiatric rehabilitation therapists help patients recognize and accept their emotions as objective realities. Patients identify triggering events and, through discussion and simulation training, put into perspective and review their emotions. They engage in debate around irrational beliefs, and re-plan and reassess the severity of situations, which helps patients realize their capability to handle difficulties and challenges. Psychiatric rehabilitation therapists remind patients not to exaggerate issues, suggesting the establishment of clear, manageable goals for planning and anticipation. This helps shift their mindset from self-blame and catastrophizing to acceptance, re-planning, and focused attention.

Positive psychological suggestions in the course help patients understand that the same event can be perceived differently by different people, with varying perspectives, viewpoints, and emotional tendencies. There is no right or wrong, but there are adaptive and maladaptive strategies. Zoromba’s study indicates that depression affects individuals’ thoughts and feelings, and emotional management training can alleviate the suffering of depression patients (35). Suddell’s research shows that online emotional training for depression patients can promote positive cognitive changes (36).

### 4.3 “Internet +” continuity emotional management training enhances social adaptability in depression patients

The study shows that post-intervention SAFE scale scores in the intervention group were (10.68 ± 1.21), significantly lower than the control group’s (15.89 ± 1.85), with a statistically significant difference (*P* < 0.05). Depression patients often exhibit emotion-related cognitive biases, such as overgeneralization and self-negation (37–41). Ezawa’s research indicates that cognitive training can enhance the social adaptability of depression patients (42), and emotional management training is a core theory in cognitive-behavioral therapy (43–45). Patients can understand cognitive biases and recognize how their thinking patterns impact their emotions, through the assistance of psychiatric rehabilitation therapists. Psychiatric rehabilitation therapists help patients assess their emotions, including identification and expression of emotions. For example, psychiatric rehabilitation therapists guide patients to articulate the events that trigger their depressive emotions and discuss in groups how they manage their emotions when feeling down. This process helps patients adjust and reshape negative cognitive biases, promoting positive cognition and self-affirmation. Patients are taught emotion regulation techniques, such as self-suggestion, attention diversion, positive communication, and interpersonal relationship building. Through role-playing and simulation exercises, patients receive emotional and communication support, enhancing their interaction skills and social adaptability. Multiple studies have shown that emotional management training in cognitive-behavioral therapy can promote the recovery of social functioning in depression patients (46–51).

## 5 Conclusion

“Internet +” continuous emotion management training can alleviate the degree of depression in post-discharge patients, enhancing their emotional management and social adaptability. The implementation of continuous rehabilitation training via “Internet +” facilitates online management of discharged depression patients, demonstrating feasibility in clinical practice. A limitation of this study is the small sample size of patients. We will collect more cases to further validate our conclusions. Additionally, the observation period for patients was short. Our team will continue to follow up with depression patients to assess the long-term effects of “Internet +” continuous emotional management training. It is recommended that hospitals form an “Internet +” intervention team to conduct online follow-ups, psychological consultations, therapy, and medication guidance, thereby standardizing the provision of continuous rehabilitation services for depression patients post-discharge, which could also be a direction for future research.

## Data Availability

The original contributions presented in the study are included in the article/supplementary material. Further inquiries can be directed to the corresponding author.
